# Validation of the UB‐ROSC Score for Predicting OHCA Survival in Chiayi City, Taiwan

**DOI:** 10.1155/emmi/5871234

**Published:** 2026-07-13

**Authors:** Wei Ta Chang, Chung Chyi Chou, Chia-Chou Tsai

**Affiliations:** ^1^ Department of Environmental and Safety Engineering, Da-Yeh University, Changhua, 510, Taiwan, dyu.edu.tw; ^2^ Chiayi City Fire Bureau, Chiayi, 600, Taiwan

**Keywords:** calibration, EMS system, external validation, OHCA, ROSC, UB-ROSC score

## Abstract

Out‐of‐hospital cardiac arrest (OHCA) is a critical public health issue, with survival rates varying widely due to multiple well‐established factors, including emergency medical service (EMS) system structure, bystander response, initial cardiac rhythm, and patient‐level characteristics. This study evaluates the Utstein‐Based Return of Spontaneous Circulation (UB‐ROSC) score’s performance in predicting sustained Return of Spontaneous Circulation (ROSC > 2 h) in a midsized Asian city where EMS delivery is standardized and geographic variation in access is minimal. A total of 209 OHCA cases from Chiayi City in 2024 were analyzed, using Utstein‐aligned data to compute UB‐ROSC scores. Predictive performance was assessed via receiver operating characteristic (ROC) curves, calibration intercept and slope, the Hosmer–Lemeshow goodness‐of‐fit test, and bootstrap internal validation, focusing on sustained return of spontaneous circulation (ROSC) (> 2 h). After excluding pediatric and traumatic cases, 209 cases were analyzed. The cohort was 60.3% male, with 47.8% witnessed arrests and 67.0% receiving bystander CPR. The UB‐ROSC score demonstrated fair discrimination, with an area under the ROC curve (AUC) of 0.78 (95% confidence interval [CI]: 0.70–0.85), stable on bootstrap and cross‐validation. The calibration slope was 0.90 (95% CI: 0.62–1.18) but the calibration intercept was +0.71 (95% CI: 0.36–1.05), and the Hosmer–Lemeshow test was significant (*χ* ^2^ = 28.6, *p* < 0.001), indicating systematic underprediction of observed survival. Risk stratification yielded a low‐risk group (*n* = 109) with a 15.6% ROSC rate, a medium‐risk group (*n* = 94) with a 48.9% rate, and a high‐risk group (*n* = 6) with an 83.3% rate. The Chiayi cohort showed a slightly higher observed ROSC rate in the low‐risk group, whereas the medium‐ and high‐risk groups fell within the predicted ranges although estimates in the high‐risk group were imprecise because of the small sample size. Local recalibration may be needed before applying absolute probability estimates in clinical settings.

## 1. Introduction

Out‐of‐hospital cardiac arrest (OHCA) remains a critical public health challenge worldwide, characterized by alarmingly low survival rates despite substantial advancements in emergency medical services (EMSs) and resuscitation science. The global incidence of EMS‐treated OHCA ranges from 30 to 97 cases per 100,000 population per year, with overall survival to hospital discharge varying widely between 2% and 20% depending on geographic, infrastructural, and sociocultural factors [1, 2]. Timely intervention is paramount in improving OHCA outcomes. The “Chain of Survival,” emphasizing early recognition, immediate cardiopulmonary resuscitation (CPR), rapid defibrillation, effective advanced life support, and integrated postcardiac arrest care, forms the cornerstone of resuscitation guidelines globally [3]. However, translating these guidelines into consistently high survival rates remains challenging, particularly in regions with heterogeneous EMS infrastructures.

In Taiwan, OHCA constitutes a significant health burden, with EMS‐treated incidence reported at approximately 58 per 100,000 population per year [4]. Over the past 2 decades, concerted efforts by the Ministry of Health and Welfare, alongside local governments, have led to substantial improvements in public CPR education, automated external defibrillator (AED) deployment, and EMS response capabilities. Because model performance is expected to vary across populations and settings, the local validation of region‐specific prognostic tools is essential before their adoption in diverse EMS systems [5]. Notably, Chiayi City has distinguished itself with proactive community engagement initiatives, continuously improving local EMS services and resulting in bystander CPR rates exceeding 60% and median EMS response times reduced to 5 min [6].

Predicting patient outcomes following OHCA is crucial for guiding prehospital and in‐hospital management, resource allocation, and informing discussions with patient families. Several prognostic models have been developed to estimate survival probabilities based on prehospital variables, among which the Utstein‐Based Return of Spontaneous Circulation (UB‐ROSC) score [7] has gained prominence for its simplicity and robust discriminative ability.

Despite its validation in European cohorts, the generalizability of the UB‐ROSC score to non‐European settings remains uncertain. Differences in EMS system structures, response protocols, public health initiatives, and cultural attitudes toward resuscitation necessitate region‐specific validations. This study aims to address this gap by evaluating the applicability of the UB‐ROSC score in predicting OHCA survival within the context of Chiayi City, Taiwan.

### 1.1. Prognostic Models in OHCA: An Overview

The development of prognostic models for OHCA outcomes has evolved significantly over the past 2 decades. Early models primarily relied on in‐hospital variables, limiting their utility in prehospital settings where critical triage decisions are made.

Among these, the Return of Spontaneous Circulation After Cardiac Arrest (RACA) score [8] was one of the first to leverage Utstein variables for return of spontaneous circulation (ROSC) prediction. The RACA score demonstrated close agreement between predicted and observed ROSC in its validation cohort; however, its complexity and reliance on multiple data points limited real‐time application. Subsequently, the Cardiac Arrest Hospital Prognosis (CAHP) score emerged, focusing on predicting neurological outcomes post‐OHCA. Although offering valuable prognostic insights, the CAHP score primarily catered to in‐hospital clinicians, underscoring the need for simpler, EMS‐friendly tools [9].

The UB‐ROSC score addressed these limitations by distilling prognostic assessment into seven readily available prehospital variables: age, gender, witnessed status, bystander CPR, initial rhythm, arrest location, and EMS response time. The introduction of Utstein‐style reporting facilitated standardized data collection, enabling the development of more accessible predictive tools based on prehospital variables. Its user‐friendly scoring system and robust performance (area under the ROC curve [AUC] 0.79 for ROSC prediction) facilitated widespread adoption across European EMS systems.

### 1.2. External Validation Challenges and Regional Adaptations

External validation is pivotal in assessing the generalizability of predictive models across diverse healthcare settings. Several studies have highlighted the challenges of applying models like UB‐ROSC beyond their development cohorts. For instance, Czapla et al. [10] validated the UB‐ROSC score in a Polish cohort, noting discrepancies in calibration despite preserved discriminative power. Similar findings were observed in Japanese validations, where differences in EMS protocols and public CPR engagement influenced model performance [11]. In the Asia‐Pacific region, EMS systems exhibit notable heterogeneity. Countries like Japan and South Korea have centralized EMS structures with high public CPR participation, while others, including parts of Southeast Asia, grapple with infrastructural limitations. Taiwan’s EMS system, mostly operated under the Fire Department framework, has evolved rapidly, with substantial investments in dispatcher‐assisted CPR (DA‐CPR) programs and AED accessibility. Studies from Singapore and South Korea have emphasized the importance of localized validation and recalibration of predictive models to account for regional variances in EMS efficiency and public health initiatives [12, 13]. These findings underscore the necessity of context‐specific evaluations before integrating predictive scores into clinical decision‐making.

### 1.3. The Need for City‐Level UB‐ROSC Validation in Chiayi City

Despite Taiwan’s strides in improving OHCA outcomes, research on predictive model validation remains limited. A recent multicenter study externally validated prehospital ROSC prediction scores, including UB‐ROSC, in a Taiwanese cohort, reporting an AUROC of 0.747 and noting calibration concerns at higher predicted probabilities [14]. While this study demonstrates UB‐ROSC’s applicability in Taiwan at a national scale, city‐level and system‐specific validations remain important for capturing the effect of local EMS infrastructure, bystander engagement, and geographic density on model performance.

Chiayi City is a densely populated urban municipality in southern Taiwan (266,000 inhabitants/60 km^2^). The single‐tiered EMS system is publicly funded and dispatched via a centralized 119 command center, which provides DA‐CPR instructions to callers. Chiayi City presents an ideal setting for such validation, given its robust EMS infrastructure, high bystander CPR rates, and commitment to continuous quality improvement. Evaluating the UB‐ROSC score in this context not only assesses its predictive accuracy but also informs potential recalibration needs. Accurate prediction of ROSC likelihood can aid in triaging resource allocation, optimizing hospital destination decisions, and facilitating transparent communication with patients’ families regarding prognosis.

### 1.4. Emerging Trends and Future Directions

Recent advancements in machine learning and artificial intelligence offer promising avenues for enhancing OHCA outcome prediction. Studies have explored the application of machine‐learning models to EMS and registry datasets for OHCA outcome prediction [15]. While these technologies hold significant potential, their complexity and resource requirements pose challenges for real‐time EMS deployment, particularly in resource‐limited settings. Consequently, simple, interpretable models like UB‐ROSC remain valuable, especially when complemented by region‐specific calibrations. Integrating such models into EMS protocols, supported by continuous validation and adjustment, aligns with the broader goal of evidence‐based, patient‐centered emergency care.

## 2. Methods

### 2.1. Study Design and Setting

This retrospective study analyzed 209 adult OHCA cases managed by the Chiayi City Fire Bureau from January to December 2024. Inclusion criteria encompassed adult patients with nontraumatic OHCA attended by EMS personnel. Data were extracted from Utstein‐aligned audit forms, capturing key variables such as age, gender, witnessed status, bystander CPR, initial rhythm, arrest location, and EMS response time.

### 2.2. Participants

Inclusion criteria are as follows: Adult patients with nontraumatic OHCA attended by EMS personnel.

Exclusion criteria are as follows:

1. Trauma: Patients with arrest secondary to blunt force, hanging, drowning, or asphyxia were excluded.
2. Pediatric cases (< 18 years): The etiology of pediatric arrest is predominantly respiratory, and the UB‐ROSC score is not calibrated for this population. One case was excluded on this basis.
3. Cases where resuscitation was not attempted due to evident death (rigor mortis).

**FIGURE 1:**
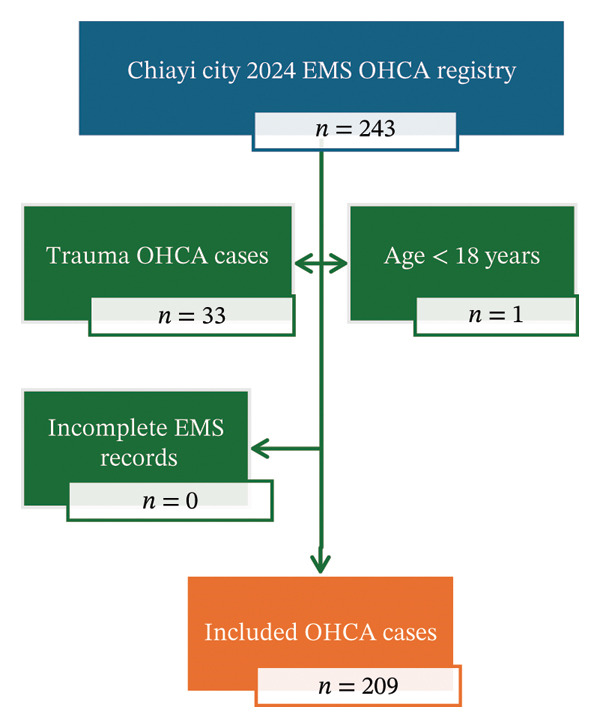
Study flowchart for selecting OHCA cases.

Of 243 cases in the 2024 EMS OHCA registry, 33 traumatic cases and 1 pediatric case were excluded, yielding 209 included cases (Figure 1). No cases had incomplete EMS records.

### 2.3. UB‐ROSC Scoring and Stratification

Data were extracted in accordance with the Utstein style. The UB‐ROSC score was calculated for each patient by summing the coefficients assigned to seven predictors: age (below/above 80 years), gender, witnessed arrest, bystander CPR, location, initial rhythm (shockable vs. nonshockable), and EMS response time, using the assigned point values from Baldi et al.’s original model (Table 1). The seven UB‐ROSC predictor variables (age, sex, witnessed status, bystander CPR, location, initial rhythm, and EMS response time) were captured prehospital and finalized on standardized Utstein‐aligned audit forms by EMS personnel at hospital handover, before the primary outcome (sustained ROSC at 2 h) was ascertained.

**TABLE 1:** UB‐ROSC score components (Baldi et al.) and scoring modifications applied in the Chiayi validation.

Predictor	Category	Score	Chiayi scoring note
Sex	Female/male	0/−3	Unchanged

Age	< 80/≥ 80	0/−9	Unchanged

Etiology	Cardiac	0	Only nontraumatic cardiac arrests included

Location	Home/Nursing home	0/−7	Nursing home includes sanatorium
	Workplace/school	6/0	Unchanged
	Street/public building	4/5	Arrest during transport coded as public/ambulance
	Sport	7	Unchanged

Bystander and CPR	No witness, no CPR	0	Unchanged
	No witness, CPR	−5	Unchanged
	Witnessed, no CPR	2	Unchanged
	Witnessed, CPR	4	Unchanged
	EMS witnessed	13	Coding rules described in §2.3

Rhythm	Nonshockable/shockable	0/21	Missing rhythm (*n* = 8) coded nonshockable

EMS response time	≤ 10/11–15/≥ 15 min	0/−4/−7	Unchanged

Constant	—	−16	Unchanged

All cases were assigned a cardiac etiology component of 0 points, consistent with our nontraumatic inclusion criterion. Two categories of cases required predefined coding rules because the original UB‐ROSC score did not include a directly corresponding category.

First, cardiac arrest witnessed by EMS personnel (*n* = 17) was coded with no‐flow time minimized: Bystander CPR = yes, Response time = 0 min, and Location = public/ambulance for the eight events occurring during transport or at the hospital interface. For EMS‐witnessed events at other locations (*n* = 9), only the Bystander CPR component was set to “witnessed with CPR.” These rules most faithfully reflect the immediate availability of professional resuscitation, which is the dominant driver of outcome in EMS‐witnessed arrest and which the original Baldi et al.’s model implicitly captures through the response‐time and bystander‐CPR components.

Second, cases with missing initial rhythm (*n* = 8; 3.8%) were classified as nonshockable. This conservative classification was based on the cohort’s prevailing nonshockable rhythm prevalence (approximately 82%) and is consistent with the imputation approach used in prior external validations. We performed a sensitivity analysis under an alternative rule (missing rhythm reclassified as shockable; see Results §3.4 and Supporting Table S3); discrimination was unchanged.

The complete dataset of 209 cases was systematically reviewed for consistency and completeness. Missing values for witness status and location were minimal (< 1%) and were cross‐referenced against the corresponding incident reports. All UB‐ROSC score calculations were verified through automated scoring scripts followed by manual spot‐checking of a 10% random sample. The final verified dataset is provided as Supporting Table S1.

The resulting scores were stratified into three risk groups (Low ≤ −19, Medium −18 to +12, and High ≥ +13). These cutoffs were derived from the external validation framework published by Caputo et al. [16], as the original Baldi et al.’s study did not explicitly define categorical thresholds. Adopting these thresholds facilitates direct comparison with benchmark data and maintains methodological consistency with prior validation studies.

### 2.4. Outcome Definition

The primary outcome was sustained ROSC for at least 2 h after arrest, confirmed through hospital records and consistent with the original Utstein‐style definition of sustained ROSC. Three considerations supported this endpoint selection.

First, sustained ROSC is the endpoint against which the UB‐ROSC score was originally developed [7] and against which the largest published external validation [16] was performed; using the same definition allows a like‐for‐like comparison of discrimination and calibration.

Second, the Chiayi City Fire Bureau registry, an EMS‐derived dataset, captures resuscitation outcomes up to and shortly after hospital handover but does not include linked hospital‐discharge or 30‐day survival data. Survival to hospital discharge—the gold‐standard OHCA outcome—was, therefore, not available for this study. We have framed our findings as a system‐specific external validation of the score against its originally intended endpoint, and we note this data‐availability constraint in the Limitations.

Third, we recognize that other validations (e.g., Fan et al. [14]) have used prehospital ROSC rather than sustained ROSC. The 2‐h threshold applied here is stricter; this difference in endpoint definition may partly explain across‐study variation in apparent calibration, and we discuss this explicitly in §4.2. Outcome status (sustained ROSC ≥ 2 h) was ascertained from prehospital and hospital handover records independently of the UB‐ROSC score, which was computed post hoc from the registry solely for the purposes of this validation.

### 2.5. Statistical Analysis and TRIPOD‐Aligned Reporting

The analysis follows the TRIPOD statement for the external validation of a multivariable prediction model (Collins et al., Ann Intern Med 2015). A completed TRIPOD checklist is provided as Supporting information S2.

#### 2.5.1. Predicted Probability Derivation

The UB‐ROSC score is a rounded integer transformation of the original Baldi et al.’s logistic regression linear predictor, with each β coefficient multiplied by ten. We, therefore, derived per‐patient predicted probability as *P* = 1/(1 + exp (−SCORE/10)). This formulation reproduces the score‐to‐probability mapping shown in the original nomogram and yields category‐level mean predictions that closely match the published Baldi probability ranges.

#### 2.5.2. Discrimination

The c‐statistic (AUC) was computed for the full cohort and within prespecified subgroups (witnessed vs. unwitnessed, civilian‐ vs. EMS‐witnessed, shockable vs. nonshockable rhythm, bystander CPR yes vs. no). 95% confidence intervals (CIs) were calculated using DeLong’s method.

#### 2.5.3. Calibration

**FIGURE 2:**
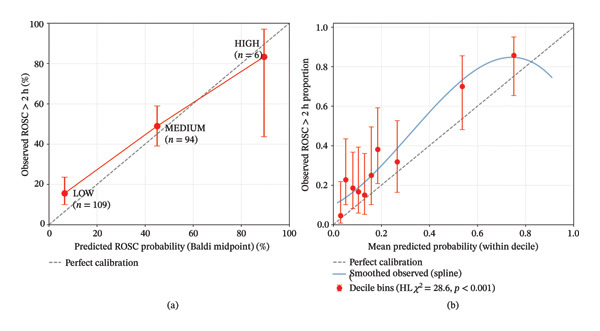
Calibration plots. (a) Stratum‐level calibration using Baldi‐predicted midpoints; vertical bars are Wilson 95% CIs. (b) Decile‐based calibration with a smoothed observed curve (Hosmer–Lemeshow *χ* ^2^ = 28.6, *p* < 0.001).

We assessed (i) the calibration intercept (calibration‐in‐the‐large), fitted as a logistic model with the linear predictor as an offset and the intercept estimated, where 0 indicates a perfectly calibrated mean prediction; (ii) the calibration slope, fitted as a logistic regression of the observed outcome on the linear predictor, where 1 indicates an ideal slope; and (iii) the Hosmer–Lemeshow goodness‐of‐fit test across deciles of predicted probability. A stratum‐level calibration plot and a decile‐level calibration plot with a smoothed observed curve are presented in Figure 2.

#### 2.5.4. Internal Validation

To quantify optimism in the apparent AUC, we conducted 1000 nonparametric bootstrap resamples and computed the optimism‐corrected AUC following Harrell. Because UB‐ROSC is applied here as a fixed external rule (no refitting), the expected optimism is near zero; the analysis serves as a robustness check. We additionally report the 10‐fold cross‐validated AUC.

#### 2.5.5. Sensitivity Analyses

We repeated the discrimination and Hosmer–Lemeshow analyses under (a) exclusion of EMS‐witnessed cases (*n* = 17), (b) exclusion of cases with imputed rhythm (*n* = 8), (c) exclusion of both, and (d) the alternative imputation of missing rhythm as shockable rather than nonshockable. Results are reported in Supporting Table S3.

#### 2.5.6. Sample Size

The cohort comprises 209 cases with 68 ROSC events. With 7 UB‐ROSC predictors, the events‐per‐variable ratio is 9.7, at the conventional borderline‐adequate threshold for external validation (Riley et al., Stat Med 2019). Achieving a 95% CI half‐width of 0.05 around the AUC would require approximately 150 events; we discuss the implications of the current sample size in §4.5.

All analyses were performed in Python (Version 3.11) using Scikit‐learn, SciPy, and Pandas. Two‐sided *p* < 0.05 was used for significance testing.

## 3. Results

### 3.1. Cohort Characteristics

Among 209 OHCA cases, 35.4% were aged ≥ 80 years and 60.3% were male. Witnessed arrests occurred in 47.8%, bystander CPR was initiated in 67.0%, and initial shockable rhythms were observed in 18.2%. The median EMS response time was 5.0 min. A total of 68 patients (32.5%) achieved sustained ROSC > 2 h. For comparison with the development cohort, Baldi et al. [7] derived UB‐ROSC in 1962 OHCAs from northern Italy and southern Switzerland (62% male; mean age 73 ± 16 years); the Chiayi sample is broadly comparable in sex distribution and age but differs most notably in a substantially higher bystander‐CPR rate (67.0%), consistent with the calibration shift described in §4.3.

### 3.2. Risk Stratification and Discrimination

UB‐ROSC score stratification yielded 109 cases in the low‐risk group (≤ −19), 94 in the medium‐risk group (−18–+12), and 6 in the high‐risk group (≥ +13). As shown in Table 2, the observed ROSC rate in the low‐risk group (15.6%) marginally exceeded the original UB‐ROSC prediction (< 12%). The medium‐risk group (48.9%) fell within the predicted 18%–72% range, toward its upper part. The high‐risk group achieved a nominally high ROSC rate of 83.3% (5/6); however, this estimate carries wide statistical uncertainty (95% CI: 38.6%–99.6%) due to the very small sample size (*n* = 6) and should not be interpreted as confirmed calibration at this stratum.

**TABLE 2:** Stratification of UB‐ROSC risk groups and sustained ROSC rates in Chiayi City compared to Baldi et al.’s European cohorts.

UB‐ROSC risk group	Chiayi cases (*n*)	ROSC > 2 h (*n*)	ROSC > 2 h rate (%)	95% CI	Baldi et al. ROSC rate	Assessment
Low (≤ −19)	109	17	15.6%	10.0%–23.6%	1%–12%	Slightly above predicted
Medium (−18 to +12)	94	46	48.9%	39.1%–58.9%	18%–72%	Within range (upper part)
High (≥ +13)	6	5	83.3%	38.6%–99.6%	80%–99%	Consistent (imprecise, *n* = 6)

*Note:* Baldi et al.’s predicted ROSC rates were derived from the nomogram reported in the original validation study. The wide 95% CI in the high‐risk group reflects the small sample size (*n* = 6) and should be interpreted with caution.

**FIGURE 3:**
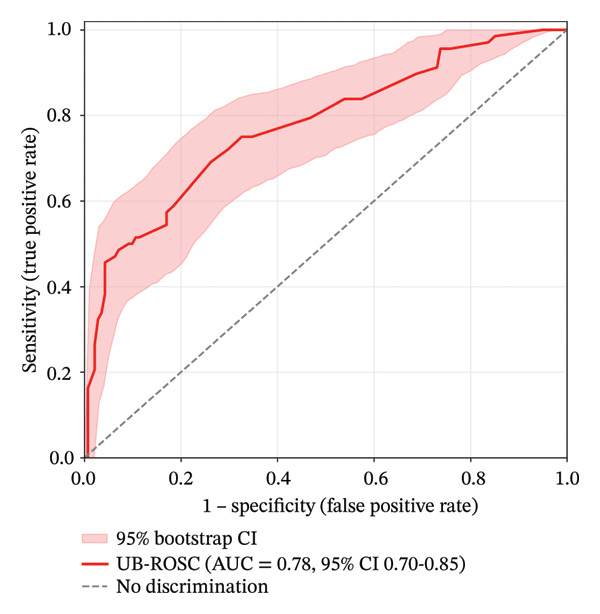
ROC curve for UB‐ROSC predicting sustained ROSC > 2 h in the Chiayi cohort (*N* = 209). AUC: 0.78 (95% CI: 0.70–0.85); shaded band shows the bootstrap 95% confidence interval.

The discrimination of the UB‐ROSC score for predicting sustained ROSC was fair, with an AUC of 0.78 (95% CI: 0.70–0.85) (Figure 3). This performance is comparable to the original derivation study.

### 3.3. Calibration Intercept, Slope, and Hosmer–Lemeshow

To complement the stratum‐level calibration shown in Figure 2A, we conducted formal calibration testing using per‐patient predicted probabilities derived from the UB‐ROSC score. The calibration intercept (calibration‐in‐the‐large) was +0.71 (95% CI: 0.36–1.05), indicating that the original model systematically underpredicts observed ROSC in this cohort: observed event rates exceed Baldi‐derived predictions on the log‐odds scale. The calibration slope was 0.90 (95% CI: 0.62–1.18), and its CI encompasses the ideal value of 1.0, indicating that the linear relationship between score and outcome on the log‐odds scale is preserved. The Hosmer–Lemeshow goodness‐of‐fit test across deciles of predicted probability was statistically significant (*χ* ^2^ = 28.6, df = 8, *p* < 0.001), confirming meaningful miscalibration of absolute predicted probabilities. Decile‐level observed and predicted probabilities are shown in Figure 2B; observed proportions exceed predicted across the lower and middle deciles, consistent with the positive calibration intercept.

Taken together, these results indicate that the UB‐ROSC linear predictor discriminates appropriately in the Chiayi cohort but that the original score‐to‐probability mapping requires local recalibration before absolute probability estimates can be used for clinical decision support.

### 3.4. Internal Validation and Subgroup Discrimination

#### 3.4.1. Internal Validation

The apparent AUC was 0.776, the bootstrap‐corrected AUC was 0.778 (optimism Δ = −0.002; bootstrap 95% percentile interval 0.700–0.839), and the 10‐fold cross‐validated AUC was 0.780 (SD 0.105). As UB‐ROSC is applied here as a fixed external rule, the near‐zero optimism indicates that the observed discrimination is not the product of refitting or overfitting on this dataset.

#### 3.4.2. Subgroup Discrimination

AUC for the witnessed subgroup (any witness, *n* = 100, 49 events) was 0.746 (95% CI: 0.649–0.843); for the unwitnessed subgroup (*n* = 109, 19 events) it was 0.662 (95% CI: 0.518–0.806). Discrimination was strongest in the civilian‐witnessed subgroup (AUC: 0.781, *n* = 82) and weakest in the small EMS‐witnessed subgroup (AUC: 0.477, *n* = 17), though the latter estimate is highly imprecise. AUC was 0.723 in shockable‐rhythm cases (*n* = 38) and 0.677 in nonshockable cases (*n* = 171) and 0.798 vs. 0.715 in bystander‐CPR‐performed vs. not‐performed subgroups. The full subgroup forest plot is provided as Supporting Figure S1.

#### 3.4.3. Sensitivity Analyses

Discrimination was robust to coding decisions: AUC was 0.766 when EMS‐witnessed cases were excluded, 0.776 when cases with imputed rhythm were excluded, 0.772 when both were excluded, and 0.786 under the alternative imputation of missing rhythm as shockable. The Hosmer–Lemeshow test remained significant across all four scenarios (all *p* < 0.02), indicating that the miscalibration of absolute probabilities is not driven by imputation choices. Full sensitivity results are provided as Supporting Table S3.

## 4. Discussion

### 4.1. Principal Findings

This study validates the predictive performance of the UB‐ROSC score in the context of Chiayi City, Taiwan. The score demonstrated fair discrimination (AUC: 0.78, 95% CI: 0.70–0.85), with stability confirmed by bootstrap optimism correction and 10‐fold cross‐validation. Calibration findings were mixed: the calibration slope of 0.90 (95% CI: 0.62–1.18) is statistically consistent with the ideal value of 1.0, but the calibration intercept of +0.71 (95% CI: 0.36–1.05) and a significant Hosmer–Lemeshow test (*χ* ^2^ = 28.6, *p* < 0.001) indicate systematic underprediction of observed survival. At stratum level, the low‐risk group’s observed ROSC (15.6%) modestly exceeded the original Baldi prediction (≤ 12%); the medium‐risk group fell within the predicted range (48.9%; predicted 18%–72%) although toward the upper part of that range; and the high‐risk group achieved 83.3% ROSC consistent with prediction although that estimate carries wide uncertainty (95% CI: 38.6%–99.6%) due to the very small sample size (*n* = 6). These patterns indicate that the UB‐ROSC linear predictor discriminates appropriately in Chiayi while its mapping to absolute probability requires local recalibration.

### 4.2. Comparison With Previous Studies

Our findings on discrimination align with the original derivation by Baldi et al. [7], who reported an AUC of 0.79 versus our 0.78. The pattern in our medium‐risk group, where observed ROSC sat toward the upper end of the predicted 18%–72% range, is consistent with findings from prior Asia‐Pacific validations (e.g., Czapla et al. [16] and Kashiura et al. [11]), in which differences in EMS protocols, response‐time distributions, and bystander engagement systematically affect score performance on the absolute‐probability scale while preserving discrimination.

Compared with Fan et al. [14], who reported an AUROC of 0.747 in a Taiwanese multicenter dataset, our single‐city cohort achieved an AUC of 0.78. The slightly higher discrimination in Chiayi may reflect the relatively homogeneous EMS environment of a single urban system with standardized protocols, versus the intersystem variability inherent in a multicenter design. Both studies similarly noted calibration challenges at higher predicted probabilities. Two further factors limit direct comparability: Fan et al. used prehospital ROSC as the endpoint, whereas we used sustained ROSC > 2 h, and their cohort had a different case mix. Differences in endpoint definition probably contribute to the observed differences in apparent calibration.

Another recent Taiwanese multicenter study by Wang et al. [15] externally validated UB‐ROSC alongside other prehospital prediction scores for neurological outcome at hospital discharge and found that UB‐ROSC performed favorably, outperforming both the prehospital‐ROSC score and the Swedish Cardiac Arrest Risk Score model. Because that study, Fan et al., and the present analysis each adopted a different primary endpoint, their absolute performance estimates are not directly comparable; taken together, however, they consistently support the discriminative validity of UB‐ROSC across diverse Taiwanese EMS settings. The present single‐city cohort adds granular, system‐specific evidence for the sustained‐ROSC endpoint and reinforces that local recalibration is required before its absolute probabilities are applied clinically.

### 4.3. Possible Explanations for Discrepancies

The most notable calibration finding is the model’s underestimation of survival among low‐risk patients (15.6% observed vs. < 12% predicted). Rather than indicating model failure, this discrepancy may reflect contextual differences between Chiayi and the European derivation cohort. Notably, the Chiayi cohort demonstrated a bystander CPR rate of 67.0% and a median EMS response time of 5.0 min, both substantially exceeding European benchmarks. While these system‐level characteristics could plausibly contribute to the observed calibration intercept, determining whether they causally drive improved outcomes would require a stratified analysis linking these metrics to individual‐level ROSC, which is beyond the scope of this external validation.

The medium‐risk group’s observed rate (48.9%) sat in the upper portion of the predicted 18%–72% range; this may suggest calibration variation but does not indicate clear underestimation by the original UB‐ROSC model in this stratum. The clearer signal of miscalibration arises in the low‐risk group, where observed ROSC exceeded the predicted ceiling and in the significantly positive calibration intercept and Hosmer–Lemeshow result for the cohort as a whole. The model remains a valid physiological discriminator, but its probability calibration reflects the specific EMS context of its derivation population rather than universal survival probabilities.

### 4.4. Clinical and Operational Implications

Despite the observed miscalibration, the UB‐ROSC score maintains utility for prehospital triage by effectively stratifying patients across risk categories. However, the position of the observed medium‐risk ROSC near the upper end of the predicted range and the significant Hosmer–Lemeshow result (*p* < 0.001) together indicate that clinical decisions should not rely on the score’s absolute probability estimates without local recalibration. We also caution that the *n* = 6 high‐risk group precludes confident inference about calibration in that stratum; the figure of 83.3% ROSC should be interpreted in light of its wide CI (38.6%–99.6%) and not used as a planning estimate. Future work should focus on model recalibration using local data, incorporating region‐specific variables (e.g., public CPR density and response‐time distributions), and exploring machine learning approaches that can adapt to local EMS characteristics.

### 4.5. Limitations

This study has several limitations. First, the single‐city design limits generalizability to other regions within Taiwan or to settings with different EMS structures. Second, the analysis was limited to a single calendar year (2024), giving a modest sample of 68 ROSC events. With 7 UB‐ROSC predictors, this represents an events‐per‐variable ratio of 9.7—at the conventional borderline‐adequate threshold for external validation—and the 95% CI width on the AUC (0.14) reflects this. Achieving a tighter interval (half‐width ≤ 0.05) would require approximately 150 events, motivating multiyear extraction in future work. The witnessed‐arrest rate of 47.8% is also relatively low for predictive model validation; although discrimination was preserved in subgroup analyses, model behavior in the unwitnessed subgroup is estimated with wider uncertainty. The high‐risk stratum (*n* = 6) is severely underpowered, and stratum‐specific calibration in that group is not reliably estimated. Third, our primary outcome was sustained ROSC > 2 h rather than survival to hospital discharge; the Chiayi City Fire Bureau registry, as an EMS‐derived dataset, does not include linked hospital‐discharge outcomes, and sustained ROSC was, therefore, selected because it is the endpoint used in the original derivation and the largest published external validation, enabling like‐for‐like comparison. Fourth, the UB‐ROSC score does not contain dedicated categories for EMS‐witnessed arrest or missing initial rhythm; our predefined coding rules for these situations are described in §2.3 and were tested in sensitivity analyses that showed discrimination to be robust. Fifth, the UB‐ROSC score is static and does not incorporate dynamic resuscitation variables or in‐hospital care quality. Finally, although our results identify local recalibration as a clear need, this study did not perform recalibration modeling; a prospective multiyear cohort with linked discharge outcomes is required to derive a Taiwan‐specific recalibrated UB‐ROSC and to evaluate it against the originally intended outcome.

## 5. Conclusion

This study demonstrates that the UB‐ROSC score achieves fair discrimination (AUC: 0.78, 95% CI: 0.70–0.85) in a Taiwanese urban EMS context, with discrimination confirmed by bootstrap internal validation and cross‐validation. However, the calibration intercept (+0.71) and Hosmer–Lemeshow goodness‐of‐fit test (*p* < 0.001) indicate that the original score‐to‐probability mapping systematically underpredicts observed survival in this cohort. Local recalibration is, therefore, required before the score is used to generate precise probability estimates in Taiwanese EMS systems. These findings support the value of city‐level and TRIPOD‐aligned validation studies and underscore the importance of adapting European‐derived prognostic models to local EMS characteristics prior to integration into clinical workflows.

## Author Contributions

Wei Ta Chang conceptualized the study, coordinated data collection from the Chiayi City Fire Bureau, managed the deidentification of the dataset, performed the statistical analysis, and drafted the manuscript. Chung Chyi Chou assisted in the statistical analysis, including ROC curve and calibration plot development, and contributed to the interpretation of results. Chia‐Chou Tsai assisted with data extraction and reviewed the manuscript for critical revisions. All authors contributed to the study design and interpreted the findings.

## Funding

This research received no external funding.

## Disclosure

All authors have approved the final version of the manuscript.

## Ethics Statement

This study analyzed a deidentified government‐maintained database collected by the Chiayi City Fire Bureau for public health and emergency medical services purposes and constitutes a secondary use of legally public information consistent with its original purpose. The project meets the criteria for exemption from formal Institutional Review Board review and waiver of individual informed consent under Taiwan’s Human Subjects Research Act and the “Scope of Human Research Cases Exempt from Ethics Review Committee Review” (Department of Health, Executive Yuan, Wei‐Shu‐Yi‐Tzu No. 1010265075/5079).

## Conflicts of Interest

The authors declare no conflicts of interest.

## Supporting Information

Additional supporting information can be found online in the Supporting Information section.

## Supporting information


**Supporting Information** Supporting Table S1: Deidentified dataset for all 209 cases, including UB‐ROSC scores and outcomes. Supporting Information S2: Completed TRIPOD checklist. Supporting Table S3: Sensitivity analyses. Supporting Figure S1: Subgroup AUC forest plot. The STROBE statement is also provided.

## Data Availability

The data that support the findings of this study are available from the corresponding author upon reasonable request.
