# Supplementary material for: Validation of the UB‐ROSC Score for Predicting OHCA Survival in Chiayi City, Taiwan
Source: Emerg Med Int. 2026 Jul 13;2026:5871234. doi: 10.1155/emmi/5871234 (PMC13359025; doi:10.1155/emmi/5871234)

Subgroup discrimination of the UB-ROSC score

Pre-specified subgroups; square size ∝ n; 95% CIs by DeLong's method. Dashed line, 0.5 (chance); dotted line, overall AUC 0.78. N = 209.

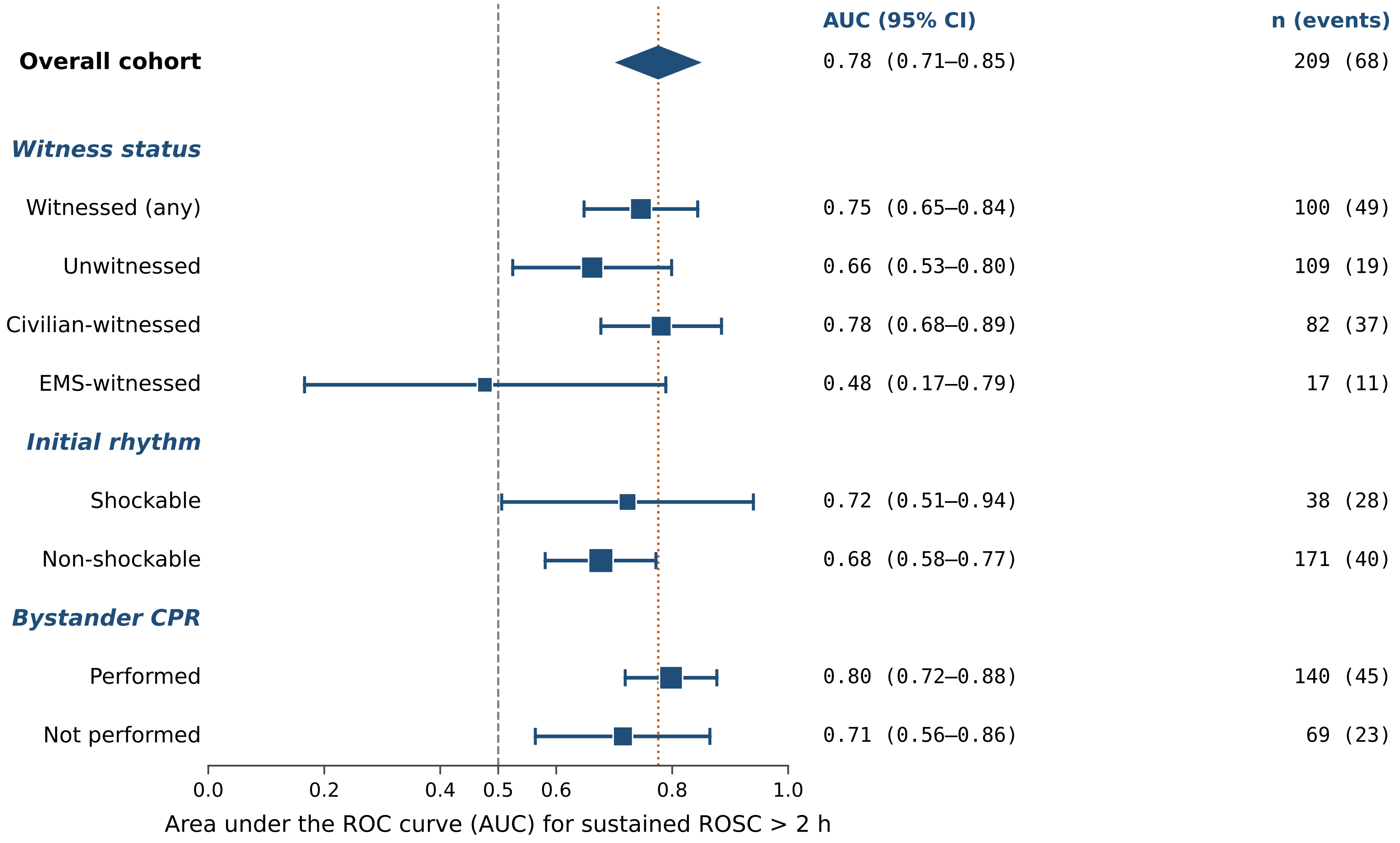

Supplement: Supplementary file 1 — Supporting Information Supporting Table S1: Deidentified dataset for all 209 cases, including UB‐ROSC scores and outcomes. Supporting Information S2: Completed TRIPOD checklist. Supporting Table S3: Sensitivity analyses. Supporting Figure S1: Subgroup AUC forest plot. The STROBE statement is also provided. [file EMMI-2026-5871234-s001.zip › Supplementary_Figure_S1_subgroup_AUC_forest.pdf]
