# Supplementary material for: Validation of the UB‐ROSC Score for Predicting OHCA Survival in Chiayi City, Taiwan
Source: Emerg Med Int. 2026 Jul 13;2026:5871234. doi: 10.1155/emmi/5871234 (PMC13359025; doi:10.1155/emmi/5871234)
Supplement: Supplementary file 1 — Supporting Information Supporting Table S1: Deidentified dataset for all 209 cases, including UB‐ROSC scores and outcomes. Supporting Information S2: Completed TRIPOD checklist. Supporting Table S3: Sensitivity analyses. Supporting Figure S1: Subgroup AUC forest plot. The STROBE statement is also provided. [file EMMI-2026-5871234-s001.zip › Supplementary_Table_S3_sensitivity.docx]

**Supplementary Table S3. Sensitivity analyses of UB-ROSC discrimination and calibration under alternative coding of EMS-witnessed and missing-rhythm cases.**

Validation of the UB-ROSC score for predicting sustained ROSC (> 2 h) in 209 out-of-hospital cardiac arrests, Chiayi City, 2024. The score was applied as a fixed external rule in every scenario; only the coding of cases without a directly corresponding UB-ROSC category was varied.

| **Scenario** | **N** | **ROSC > 2 h, n** | **AUC (95% CI)** | **Hosmer–Lemeshow χ² (df = 8)** | **p** |
| --- | --- | --- | --- | --- | --- |
| **Base case — full cohort, primary coding** | 209 | 68 | 0.776 (0.706–0.846) | 28.6 | < 0.001 |
| (a) EMS-witnessed cases excluded (n = 17) | 192 | 57 | 0.766 (0.688–0.844) | 22.7 | 0.004 |
| (b) Missing-rhythm cases excluded (n = 8) | 201 | 62 | 0.776 (0.703–0.849) | 20.1 | 0.010 |
| (c) EMS-witnessed and missing-rhythm cases excluded | 188 | 54 | 0.772 (0.693–0.851) | 18.3 | 0.019 |
| (d) Missing rhythm imputed as shockable | 209 | 68 | 0.786 (0.718–0.855) | 20.0 | 0.010 |

**Notes.**

Discrimination (AUC) was computed with 95% confidence intervals by DeLong’s method. Calibration was assessed with the Hosmer–Lemeshow goodness-of-fit test across deciles of predicted probability (df = 8); per-patient predicted probabilities were derived as P = 1 / (1 + exp(−score/10)).

Scenarios (a)–(c) are complete-case exclusions; scenario (d) re-imputes the eight cases (3.8%) with missing initial rhythm as shockable rather than non-shockable (its base-case coding). Discrimination was essentially unchanged across all scenarios (AUC 0.77–0.79), and the Hosmer–Lemeshow test remained statistically significant in every scenario (all p < 0.02), indicating that the miscalibration of absolute predicted probabilities is not an artefact of the coding rules for EMS-witnessed or missing-rhythm cases.

*AUC, area under the receiver-operating-characteristic curve; CI, confidence interval; df, degrees of freedom; EMS, emergency medical services; ROSC, return of spontaneous circulation.*
