# Supplementary material for: Validation of the UB‐ROSC Score for Predicting OHCA Survival in Chiayi City, Taiwan
Source: Emerg Med Int. 2026 Jul 13;2026:5871234. doi: 10.1155/emmi/5871234 (PMC13359025; doi:10.1155/emmi/5871234)
Supplement: Supplementary file 1 — Supporting Information Supporting Table S1: Deidentified dataset for all 209 cases, including UB‐ROSC scores and outcomes. Supporting Information S2: Completed TRIPOD checklist. Supporting Table S3: Sensitivity analyses. Supporting Figure S1: Subgroup AUC forest plot. The STROBE statement is also provided. [file EMMI-2026-5871234-s001.zip › TRIPOD_Checklist_S2.docx]

**Supplementary Material S2. Completed TRIPOD Checklist**

*Validation of the UB-ROSC Score for Predicting OHCA Survival in Chiayi City, Taiwan*

**Reporting guideline.** This study is reported in accordance with the TRIPOD statement (Collins GS, Reitsma JB, Altman DG, Moons KGM. *Ann Intern Med*. 2015;162:55–63). It is an **external validation** of a previously developed multivariable prediction model (the UB-ROSC score; Baldi et al., 2020), applied as a fixed rule without refitting.

**How to read the designations.** Each item is labelled **D** (relevant to model development only), **V** (relevant to validation only), or **D;V** (relevant to both), per the original TRIPOD checklist. Development-only (D) items are marked **Not applicable** for this validation study; where the manuscript nonetheless provides the information, the location is noted.

**Locations.** Cross-references use the manuscript’s section numbers (§), tables, and figures.

| **Item** | **TRIPOD checklist item** | **Location in manuscript** | **Status and comment** |
| --- | --- | --- | --- |
| **Title and Abstract** | | | |
| **1** D;V | Identify the study as developing and/or validating a multivariable prediction model, the target population, and the outcome to be predicted. | Title page | **Addressed.** Title identifies an external validation of the UB-ROSC score, the target population (OHCA patients in Chiayi City, Taiwan), and the predicted outcome (survival / sustained ROSC). |
| **2** D;V | Provide a summary of objectives, study design, setting, participants, sample size, predictors, outcome, statistical analysis, results, and conclusions. | Abstract | **Addressed.** Abstract reports the objective, retrospective registry design, setting (Chiayi City, 2024), 209 cases, the UB-ROSC predictors, the outcome (sustained ROSC >2 h), the statistical methods (ROC, calibration intercept/slope, Hosmer–Lemeshow, bootstrap), the results, and the conclusion. |
| **Introduction** | | | |
| **3a** D;V | Explain the medical context (including whether diagnostic or prognostic) and rationale for developing or validating the model, including references to existing models. | §1, §1.1–1.2 | **Addressed.** Prognostic context of OHCA outcome prediction and the rationale for external validation are established, with references to existing models (RACA, CAHP, UB-ROSC). |
| **3b** D;V | Specify the objectives, including whether the study describes the development or validation of the model or both. | §1, §1.3 | **Addressed.** Objective is stated explicitly as external validation (not development) of the UB-ROSC score in the Chiayi City EMS system. |
| **Methods** | | | |
| **4a** D;V | Describe the study design or source of data, separately for the development and validation data sets, if applicable. | §2.1 | **Addressed.** Validation data source described as the Chiayi City Fire Bureau OHCA registry (retrospective). The development data set is identified through Baldi et al. |
| **4b** D;V | Specify the key study dates, including start of accrual, end of accrual, and, if applicable, end of follow-up. | §2.1, §2.4 | **Addressed.** Accrual period given as January–December 2024; the outcome (sustained ROSC) is assessed at ≥2 h after arrest. |
| **5a** D;V | Specify key elements of the study setting (e.g., primary care, secondary care, general population) including number and location of centres. | §1.3, §2.1 | **Addressed.** Single-tiered, publicly funded EMS dispatched via a centralized 119 command centre; a single city / single EMS system is specified. |
| **5b** D;V | Describe eligibility criteria for participants. | §2.2 | **Addressed.** Inclusion (adult non-traumatic EMS-attended OHCA) and exclusion criteria (trauma, pediatric, evident death) are specified. |
| **5c** D;V | Give details of treatments received, if relevant. | §1.3, §2.3 | **Addressed.** Relevant prehospital treatment is described (bystander and dispatcher-assisted CPR, EMS resuscitation), including coding rules for EMS-witnessed arrest. |
| **6a** D;V | Clearly define the outcome that is predicted by the prediction model, including how and when assessed. | §2.4 | **Addressed.** Primary outcome defined as sustained ROSC ≥2 h, confirmed through hospital records, with timing and ascertainment described and justified. |
| **6b** D;V | Report any actions to blind assessment of the outcome to be predicted. | §2.4 | **Addressed.** Outcome ascertainment is stated to be independent of the score: sustained ROSC (≥2 h) was determined from prehospital and hospital handover records, with the UB-ROSC score computed post hoc from the registry solely for this validation. |
| **7a** D;V | Clearly define all predictors used in developing or validating the model, including how and when they were measured. | §2.3, Table 1 | **Addressed.** All seven predictors are defined with categories, point values, and measurement source (Utstein-aligned audit forms) in Table 1. |
| **7b** D;V | Report any actions to blind assessment of predictors for the outcome and other predictors. | §2.3 | **Addressed.** Predictor assessment is stated to precede outcome ascertainment: the seven UB-ROSC variables were captured prehospital and finalized on standardized Utstein-aligned audit forms at hospital handover, before sustained-ROSC status at 2 h was known. |
| **8** D;V | Explain how the study size was arrived at. | §2.5 (Sample size), §4.5 | **Addressed.** Sample size justified: 209 cases with 68 events, events-per-variable 9.7 (Riley et al.), with explicit discussion of the resulting precision (CI half-width) on the AUC. |
| **9** D;V | Describe how missing data were handled (e.g., complete-case, single or multiple imputation) with details of any imputation method. | §2.3, §3.4 | **Addressed.** Missing rhythm (n=8) coded non-shockable with a pre-specified sensitivity analysis under the alternative coding; witness/location missingness <1% cross-referenced to incident reports. |
| **10a** D | Describe how predictors were handled in the analyses. | §2.3 (context only) | **Not applicable (development item).** Development-only item. Not applicable to an external validation; predictor coding used for scoring is nonetheless described in §2.3. |
| **10b** D | Specify type of model, all model-building procedures (including any predictor selection), and method for internal validation. | — | **Not applicable (development item).** Development-only item. The model is applied as a fixed external rule without refitting or predictor selection, so this does not apply. |
| **10c** V | For validation, describe how the predictions were calculated. | §2.5 (Predicted probability derivation) | **Addressed.** Per-patient predicted probability derivation is given explicitly: P = 1 / (1 + exp(−SCORE/10)), reproducing the original score-to-probability mapping. |
| **10d** D;V | Specify all measures used to assess model performance and, if relevant, to compare multiple models. | §2.5 | **Addressed.** Discrimination (AUC with DeLong CIs) and calibration (calibration-in-the-large intercept, calibration slope, Hosmer–Lemeshow) are pre-specified. |
| **10e** V | Describe any model updating (e.g., recalibration) arising from the validation, if done. | §4.4–4.5 | **Not applicable (not done).** No model updating or recalibration was performed; the need for local recalibration is identified as future work. |
| **11** D;V | Provide details on how risk groups were created, if done. | §2.3 | **Addressed.** Risk-group thresholds specified (Low ≤−19; Medium −18 to +12; High ≥+13) with their source (Caputo et al., 2024). |
| **12** V | For validation, identify any differences from the development data in setting, eligibility criteria, outcome, and predictors. | §1.2, §2.3, §2.4, §4.2 | **Addressed.** Differences from the European development cohort are identified: single-city Taiwanese EMS setting, predictor-coding adaptations for EMS-witnessed arrest and missing rhythm; the outcome definition is kept identical for like-for-like comparison. |
| **Results** | | | |
| **13a** D;V | Describe the flow of participants through the study, including the number with and without the outcome and, if applicable, follow-up time. A diagram may be helpful. | §2.2, Figure 1, §3.1 | **Addressed.** Participant flow reported (243 screened → 34 excluded → 209 included) with a flow diagram (Figure 1); 68 of 209 achieved the outcome. |
| **13b** D;V | Describe the characteristics of the participants (basic demographics, clinical features, available predictors), including the number with missing data. | §3.1, §2.3 | **Addressed.** Cohort characteristics reported (age, sex, witnessed status, bystander CPR, shockable rhythm, response time); predictor missingness is reported. |
| **13c** V | For validation, show a comparison with the development data of the distribution of important variables (demographics, predictors, and outcome). | Table 2, §3.1, §4.2 | **Addressed.** Stratum-level ROSC rates are compared with Baldi et al. (Table 2), and §3.1 now adds a direct comparison of the development cohort (1,962 OHCAs; 62% male; mean age 73±16 y) against the Chiayi sample, noting the markedly higher bystander-CPR rate (67.0%) as relevant to the calibration shift. |
| **14a** D | Specify the number of participants and outcome events in each analysis. | §3.4 (reported) | **Not applicable (development item).** Development-only item. Not applicable; event counts per subgroup analysis are nonetheless reported in §3.4. |
| **14b** D | If done, report the unadjusted association between each candidate predictor and outcome. | — | **Not applicable (development item).** Development-only item (candidate-predictor screening). Not applicable to an external validation of a fixed model. |
| **15a** D | Present the full prediction model to allow predictions for individuals (all regression coefficients, and model intercept or baseline survival). | Table 1, §2.5 | **Not applicable (development item).** Development-only item. Not applicable; for transparency the validated model's points and constant (Baldi et al.) are reproduced in Table 1 and the probability formula in §2.5. |
| **15b** D | Explain how to use the prediction model. | Table 1, §2.5 | **Not applicable (development item).** Development-only item. Not applicable; scoring and the score-to-probability mapping are nonetheless provided. |
| **16** D;V | Report performance measures (with CIs) for the prediction model. | §3.2–3.4, Figures 2–3, Table 2 | **Addressed.** Performance reported with CIs: AUC 0.78 (0.70–0.85); calibration intercept +0.71 (0.36–1.05); slope 0.90 (0.62–1.18); Hosmer–Lemeshow χ²=28.6, p<0.001; plus bootstrap, cross-validated, and subgroup AUCs. |
| **17** V | If done, report the results from any model updating (i.e., model specification, model performance). | — | **Not applicable (not done).** No model updating was performed, so there are no updating results to report (consistent with item 10e). |
| **Discussion** | | | |
| **18** D;V | Discuss any limitations of the study (such as nonrepresentative sample, few events per predictor, missing data). | §4.5 | **Addressed.** Limitations discussed: single-city design, single-year/modest event count (EPV 9.7), low witnessed-arrest rate, underpowered high-risk stratum (n=6), outcome limited to sustained ROSC rather than discharge, score-coding limitations, and the static nature of the score. |
| **19a** V | For validation, discuss the results with reference to performance in the development data, and any other validation data. | §4.2 | **Addressed.** Results compared with the development study (Baldi AUC 0.79 vs 0.78 here) and with other validations (Fan et al.; Czapla et al.; Kashiura et al.). |
| **19b** D;V | Give an overall interpretation of the results, considering objectives, limitations, results from similar studies, and other relevant evidence. | §4.1, §4.3 | **Addressed.** Overall interpretation provided: preserved discrimination but systematic underprediction of absolute probabilities, requiring local recalibration before clinical use. |
| **20** D;V | Discuss the potential clinical use of the model and implications for future research. | §4.4 | **Addressed.** Clinical use (triage utility, caution against using absolute probabilities) and future directions (local recalibration, region-specific variables, machine learning) are discussed. |
| **Other Information** | | | |
| **21** D;V | Provide information about the availability of supplementary resources, such as study protocol, web calculator, and data sets. | Supplementary Material | **Addressed.** Supplementary resources listed: de-identified dataset (S1), this completed TRIPOD checklist (S2), sensitivity analyses (S3), subgroup AUC forest plot (Figure S1), and the STROBE statement. |
| **22** D;V | Give the source of funding and the role of the funders for the present study. | Funding; Conflict of Interest | **Addressed.** Funding stated (“no external funding”) and conflicts of interest declared. |

**Summary:** All TRIPOD items applicable to an external validation study are addressed. Six development-only items (10a, 10b, 14a, 14b, 15a, 15b) and two model-updating items (10e, 17) are not applicable because no model was developed or recalibrated in this study; where the manuscript nonetheless reports the relevant information, the location is noted.
