# Supplementary material for: Validation of the UB‐ROSC Score for Predicting OHCA Survival in Chiayi City, Taiwan
Source: Emerg Med Int. 2026 Jul 13;2026:5871234. doi: 10.1155/emmi/5871234 (PMC13359025; doi:10.1155/emmi/5871234)
Supplement: Supplementary file 1 — Supporting Information Supporting Table S1: Deidentified dataset for all 209 cases, including UB‐ROSC scores and outcomes. Supporting Information S2: Completed TRIPOD checklist. Supporting Table S3: Sensitivity analyses. Supporting Figure S1: Subgroup AUC forest plot. The STROBE statement is also provided. [file EMMI-2026-5871234-s001.zip › STROBE-checklist-v4-cohort_2.doc]

STROBE Statement—Checklist of items that should be included in reports of ***cohort studies***

|  | Item No | Recommendation | Where Addressed in Manuscript |
| --- | --- | --- | --- |
| **Title and abstract** | 1 | (*a*) Indicate the study’s design with a commonly used term in the title or the abstract | Title (p. 0) and Abstract (p. 1). Also Methods (Section 2.1, p. 5). |
|  |  | (*b*) Provide in the abstract an informative and balanced summary of what was done and what was found | Abstract (p. 1). Includes outcomes (AUC 0.78, risk stratification results). |
| Introduction | | |  |
| Background/rationale | 2 | Explain the scientific background and rationale for the investigation being reported | Introduction (pp. 2–4). Discusses OHCA burden, Taiwan's EMS context, and need for validation. |
| Objectives | 3 | State specific objectives, including any prespecified hypotheses | Introduction (p. 3). Objective: Evaluate UB-ROSC applicability in Chiayi City. |
| Methods | | |  |
| Study design | 4 | Present key elements of study design early in the paper | Methods (Section 2.1, p. 5). Retrospective study of EMS registry data. |
| Setting | 5 | Describe the setting, locations, and relevant dates, including periods of recruitment, exposure, follow-up, and data collection | Methods (Section 2.1, p. 5). Chiayi City Fire Bureau, Jan–Dec 2024. |
| Participants | 6 | (*a*) Give the eligibility criteria, and the sources and methods of selection of participants. Describe methods of follow-up | Methods (Section 2.2, p. 5) & Figure 1 (p. 5). Inclusion: EMS-treated non-traumatic OHCA. Exclusion: Trauma, Age <18, DNAR. |
|  |  | (*b*) For matched studies, give matching criteria and number of exposed and unexposed | Not applicable. |
| Variables | 7 | Clearly define all outcomes, exposures, predictors, potential confounders, and effect modifiers. Give diagnostic criteria, if applicable | Methods (Sections 2.3 & 2.4, pp. 6–8). Predictors (Utstein variables), Outcome (ROSC >2h). |
| Data sources/ measurement | 8* | For each variable of interest, give sources of data and details of methods of assessment (measurement). Describe comparability of assessment methods if there is more than one group | Methods (Section 2.1 & 2.3, pp. 5–6). Data from Utstein-aligned EMS audit forms; scores calculated per Baldi et al. definitions. |
| Bias | 9 | Describe any efforts to address potential sources of bias | Methods (Section 2.2, p. 5). Strict exclusion criteria (trauma/pediatric) to align with original model scope. |
| Study size | 10 | Explain how the study size was arrived at | Methods (Figure 1, p. 5). All eligible cases in the 2024 registry were included (N=209) |
| Quantitative variables | 11 | Explain how quantitative variables were handled in the analyses. If applicable, describe which groupings were chosen and why | Methods (Section 2.3, pp. 6-7). UB-ROSC scores stratified into Low (-19), Medium (-18 to +12), High (+13) based on Caputo et al. (2024). |
| Statistical methods | 12 | (*a*) Describe all statistical methods, including those used to control for confounding | Methods (Section 2.4, p. 8). ROC analysis (AUC), Calibration plots, Wilson score intervals for CIs. |
|  |  | (*b*) Describe any methods used to examine subgroups and interactions | Methods (Section 2.3, p. 7) & Results (Table 2, p. 9). Stratification by risk group (Low/Medium/High). |
|  |  | (*c*) Explain how missing data were addressed | Methods (Section 2.3, p. 6). Missing rhythms () coded as non-shockable; EMS-witnessed arrests imputed to minimize no-flow bias. |
|  |  | (*d*) If applicable, explain how loss to follow-up was addressed | Figure 1 (p. 5). No loss to follow-up reported for the primary outcome (prehospital ROSC) as all data is from EMS records. |
|  |  | (*e*) Describe any sensitivity analyses | N/A. No specific sensitivity analysis performed beyond main stratification. |
| Results | | |  |
| Participants | 13* | (a) Report numbers of individuals at each stage of study—eg numbers potentially eligible, examined for eligibility, confirmed eligible, included in the study, completing follow-up, and analysed | Figure 1 (p. 5). Screened (n=243) Excluded (n=34) Included (n=209) |
|  |  | (b) Give reasons for non-participation at each stage | Figure 1 (p. 5). Trauma (), Age <18 (n=1). |
|  |  | (c) Consider use of a flow diagram | Figure 1 (p. 5). Study Flowchart included. |
| Descriptive data | 14* | (a) Give characteristics of study participants (eg demographic, clinical, social) and information on exposures and potential confounders | Results (p. 9). Age 80 (35.4%), Male (60.3%), Bystander CPR (67.0%), etc. |
|  |  | (b) Indicate number of participants with missing data for each variable of interest | Methods (Section 2.3, p. 6). Explicitly mentions missing rhythm data (). |
|  |  | (c) Summarise follow-up time (eg, average and total amount) | Methods (Section 2.4, p. 8). Outcome is immediate sustained ROSC (>2h); long-term follow-up not applicable. |
| Outcome data | 15* | Report numbers of outcome events or summary measures over time | Results (p. 9). Total ROSC >2h (n=68, 32.5%). |
| Main results | 16 | (*a*) Give unadjusted estimates and, if applicable, confounder-adjusted estimates and their precision (eg, 95% confidence interval). Make clear which confounders were adjusted for and why they were included | Table 2 (p. 9). Observed rates with 95% CIs for each risk group. AUC = 0.78 (95% CI 0.70–0.84). |
|  |  | (*b*) Report category boundaries when continuous variables were categorized | Methods (Section 2.3, p. 7). Cut-offs: Low -19, Medium -18 to +12, High +13. |
|  |  | (*c*) If relevant, consider translating estimates of relative risk into absolute risk for a meaningful time period | Discussion (Section 4.1, p. 12). Discusses absolute risk (observed %) vs predicted risk. |
| Other analyses | 17 | Report other analyses done—eg analyses of subgroups and interactions, and sensitivity analyses | Results (Table 2, p. 9). Detailed comparison of Chiayi outcomes vs. Baldi et al. predictions. |
| Discussion | | |  |
| Key results | 18 | Summarise key results with reference to study objectives | Discussion (Section 4.1, p. 12). Confirms validation (AUC 0.78) but notes calibration differences. |
| Limitations | 19 | Discuss limitations of the study, taking into account sources of potential bias or imprecision. Discuss both direction and magnitude of any potential bias | Discussion (Section 4.5, p. 13). Notes small sample size in high-risk group (n=6), single-center design, and use of ROSC proxy. |
| Interpretation | 20 | Give a cautious overall interpretation of results considering objectives, limitations, multiplicity of analyses, results from similar studies, and other relevant evidence | Discussion (Section 4.3, p. 12). Interprets "survival dividend" due to high bystander CPR; suggests need for recalibration. |
| Generalisability | 21 | Discuss the generalisability (external validity) of the study results | Discussion (Section 4.2 & 4.5, pp. 12–13). Compares with Asian/European cohorts; notes limits of single-city data. |
| Other information | | |  |
| Funding | 22 | Give the source of funding and the role of the funders for the present study and, if applicable, for the original study on which the present article is based | Funding (p. 14). No external funding received. |

*Give information separately for exposed and unexposed groups.

**Note:** An Explanation and Elaboration article discusses each checklist item and gives methodological background and published examples of transparent reporting. The STROBE checklist is best used in conjunction with this article (freely available on the Web sites of PLoS Medicine at http://www.plosmedicine.org/, Annals of Internal Medicine at http://www.annals.org/, and Epidemiology at http://www.epidem.com/). Information on the STROBE Initiative is available at http://www.strobe-statement.org.
